# Primary Human Fibroblasts in Culture Switch to a Myofibroblast-Like Phenotype Independently of TGF Beta

**DOI:** 10.3390/cells8070721

**Published:** 2019-07-13

**Authors:** Ulrike Baranyi, Birgitta Winter, Alfred Gugerell, Balazs Hegedus, Christine Brostjan, Günther Laufer, Barbara Messner

**Affiliations:** 1 Cardiac Surgery Research Laboratory, Department of Surgery, Medical University of Vienna, 1090 Vienna, Austria; 2 Department of Surgery, Medical University of Vienna, 1090 Vienna, Austria; 3 Department of Cardiology, Medical University of Vienna, 1090 Vienna, Austria; 4 Division of Thoracic Surgery, Department of Surgery, Comprehensive Cancer Center Vienna, Medical University of Vienna, 1090 Vienna, Austria; 5 Department of Cardiac Surgery, Medical University of Vienna, 1090 Vienna, Austria

**Keywords:** fibroblast, myofibroblast phenotype, alpha smooth muscle actin, CD90, ascorbic acid, procollagen I

## Abstract

Fibroblasts are the prevalent cell type and main source for extracellular matrix (ECM) in connective tissue. Depending on their origin, fibroblasts play a central role in non-pathological tissue remodeling and disease like fibrosis. This study examined the effect of established culture conditions of primary human fibroblasts, from different origins on the myofibroblast-like phenotype formation. We isolated primary human fibroblasts from aortic adventitia, lung, juvenile- and adult skin and investigated the expression levels of CD90, alpha smooth muscle actin (αSMA) and procollagen I under different concentrations of fetal calf serum (FCS) and ascorbic acid (AA) in culture media by immunoblot and immunofluorescence assays. Furthermore, we determined the viability using XTT and migration/wound healing in scratch assays. Collagen 1 secretion was quantified by specific ELISA. Primary human fibroblasts show in part a myofibroblast-like phenotype even without addition of FCS. Supplemented AA reduces migration of cultured fibroblasts with no or low concentrations of FCS. Furthermore, AA and higher concentrations of FCS in culture media lead to higher levels of collagen 1 secretion instead of procollagen I accumulation. This study provides evidence for a partial switch of primary human fibroblasts of different origin to a myofibroblast-like phenotype under common culture conditions.

## 1. Introduction

Fibroblasts represent a complex and versatile group of cells involved in many cellular processes in connective tissue and are derived from embryonic mesoderm. As such, they can be found in almost every tissue and organ of the body [1].

Fibroblasts are the primary cells that produce and maintain the extracellular matrix (ECM) in connective tissues [2]. Beyond production of ECM (e.g., different collagen types) and ground substance (e.g., glycosaminoglycans), fibroblasts play various roles in health and disease. Fibroblasts are involved in key processes such as tissue remodeling and wound healing [3]. Furthermore, fibroblasts are central mediators in inflammation, angiogenesis, cancer, and in physiological and pathological tissue fibrosis [4]. Fibrosis can affect almost every tissue of the body and is a frequent pathological feature of chronic inflammatory diseases. During fibrosis many cellular processes and different cell types are involved, specifically fibroblasts, who become activated and change their phenotype. One critical pathway in fibrosis is the transforming growth factor beta (TGF-β)-pathway [5,6].

The term myofibroblast was first described for granulation tissue in wound healing and was characterized by fibroblast induction of microfilaments and bundles similar to smooth muscle cells (SMCs) [7,8]. Later on, the involvement of myofibroblasts became recognized in different organ disorders and organ remodeling [9].

The fibroblast to myofibroblast transition involves stress fiber formation, consisting of alpha smooth muscle actin (αSMA), an actin isoform, usually expressed in vascular SMCs (VSMCs), further leading to migration, proliferation, and production of ECM, such as collagen type 1 [10]. Additional to regulators such as TGF-β, the secreted SPARC related modula calcium-binding protein 2 (SMOC2) was described to be an important element of myofibroblast transition in kidney fibrosis [11]. Increasing matrix stiffness, a phenomenon observed in aging tissue, can also drive myofibroblast activation [12,13]. Alpha SMA was demonstrated to be upregulated in rodent fibroblasts when they become activated and change their contractibility and migration pattern [14,15]. Although fibroblasts are the principal effector cells in fibrosis, macrophages and other inflammatory cells, epithelial cells, cytokines, and growth factors constitute an intricate network, which regulates myofibroblast differentiation [16].

Initiation of myofibroblast differentiation is regulated mainly by TGF-β1 and beside αSMA, also collagen type I is induced [3,16,17,18]. The addition of AA to human dermal fibroblast culture together with TGF-β1 was reported to induce a switch to a myofibroblast phenotype independently of Smad2/3 signaling [19]. AA was demonstrated to be necessary for procollagen processing and secretion in rodent cardiac fibroblasts and is connected to fibrosis [6,20].

CD90 (Thy-1) is expressed on the surface of several fibroblast subsets. The role of CD90 in myofibroblast differentiation is unclear, but CD90 may play a role in wound repair and fibrosis [21]. CD90 is a small surface glycoprotein, which was demonstrated to control proliferation and differentiation in fibroblasts. As such, activated fibroblasts express CD90. Serum as a supplement added to cell culture media is important for growth and viability of various cells and cell lines. Although serum is mostly heat inactivated, it contains many different growth factors, chemokines, cytokines, and proteins [22].

Aortic adventitial fibroblasts (AoAF) may be involved in the development of aortic diseases like aortic aneurysms and atherosclerotic vessel remodeling [23,24,25]. Therefore, to study AoAF into detail, the culture and comparison of primary human fibroblasts from various organs is of interest. In this study, we investigated the influence of usual supplements to cell culture, fetal calf serum (FCS) and the antioxidant AA on the fibroblast-to-myofibroblast differentiation of cultured primary human fibroblasts.

## 2. Material and Methods

### 2.1. Isolation of Primary Fibroblasts

Fibroblasts were isolated from the following tissues: aortic fibroblast from the ascending aorta of recipients of heart transplantation [26], lung fibroblasts from tumor-free lung tissue specimens [27], juvenile skin fibroblasts from foreskin [28], and dermal skin from tissue from healthy individuals [29]. All subjects gave written informed consent. The studies were approved by the ethics committee of the Medical University of Vienna and the General Hospital Vienna (EK 957/2011, EK 1123/2009, EK07 176-VK, EK 1280/2015).

### 2.2. Purification of Aortic Adventitial Fibroblasts and Juvenile Dermal Fibroblasts

AoAF were isolated by separating the adventitia from the media of aortic tissue. Then, adventitial tissue was incubated with collagenase/elastase mixture for 3 h at 37 °C, centrifuged and re-suspended in DMEM media (Lonza) containing 10% FCS. Isolated cells were cultured on gelatin (0.2%) coated cell culture flasks. Cells were further purified using anti-CD90 antibody-(AS02, DIA 100, Dianova, Hamburg, Germany) loaded magnetic beads (Cellection Pan mouse IgG kit Dynal, Invitrogen, Carlsbad, CA, USA) as described in [28]. Briefly, cells were treated with trypsin and incubated with CD90 beads in RPMI (containing antibiotics and 0.1% FCS). Beads bound cells were isolated with a magnetic particle concentrator (Dynal MPC-S, Dynal Invitrogen) and cultured for further analysis.

### 2.3. Culture of Primary Fibroblasts

For analysis, all primary fibroblast types were used at passages 5 to 10 and cell culture plates were coated with 0.2% gelatin (Sigma Aldrich, St. Louis, MO, USA) in PBS. Fibroblasts were grown in DMEM with 4.5 g/L glucose and glutamine (Lonza), 25 mM HEPES (Sigma Aldrich, pH 7.4), 100 µg/mL streptomycin, 100 U/mL penicillin (Pen/Strep), and 10% FCS (Linaris, LaborChemie, Vienna, Austria). After serum starvation, cells were incubated with different concentrations of heat inactivated FCS. AA (Sigma Aldrich) was added to cell culture at a final concentration of 100µM as described in [30]. Different concentrations of human serum albumin (HSA; Kedrion, Lucca, Italy) or bovine serum albumin (BSA; Sigma) were used for culture. Albumin was used in increasing concentrations of 0.0115%, 0.023%, 0.046%, 0.092%, 0.23% and 0.46% (corresponding to 0.5%, 1%, 2%, 4%, 10% and 20% FCS) Albumin concentrations used were calculated by using the albumin content in serum which is described at an amount of 23 g/L [31]. Cells were incubated for 48h before analysis and media was changed after 24 h. All experiments were repeated at least 4 times. One cell line was used for each source, with the exception of the AoAFs, which came from four different donors.

### 2.4. Scratch Assays

The wound scratch or migration assay was performed with different fibroblast types according to [32]. Cells were FCS starved for 24 h (as described above). Briefly, the cell monolayer was scraped in a straight line with a 200 µL pipet tip. Along the scratch prefixed points were selected for taking representative images using a phase-contrast microscope (Olympus, Tokyo, Japan) or using a TECAN SPARK 20M (TECAN, Morrisville, NC, USA). Wound closure or cell migration was calculated as the percentage of newly by fibroblasts covered area (3–4images per sample) in relation to the untreated control (taken as 100%). Alternatively, the cell migration was calculated by measuring cell confluence using TECAN SPARK 20M in relation to the untreated control as described above. After scratching, the medium was replaced with fresh media containing different concentrations of FCS or albumin or FCS and AA (100 µM) and cells were incubated for 18–48 h before analysis.

### 2.5. Immunofluorescence Microscopy

Fibroblasts (10^4^ per well) were seeded on 0.2% gelatin-coated 8 well glass chamber slides (Corning, Thermo Scientific, Waltham, MA, USA) and cultured in DMEM with 25 mM HEPES and Pen/Strep. Cells were incubated with different concentrations of FCS or albumin as described above. Cells were fixed with 4% paraformaldehyde, permeabilized with 0.5% saponine and unspecific binding sites were blocked using goat serum. Monoclonal antibodies were incubated at 4 °C. The following antibodies were used: anti-human fibroblast surface protein clone (FSP1) mIgM (Sigma Aldrich 1B10; 1:500), mouse-anti-human CD90 (Dianova AS02; 1:200), rabbit anti human αSMA (Abcam, Cambridge, UK, 1:100), rabbit anti-human vimentin (Santa Cruz, Dallas, TX, USA, 1:100). As secondary antibodies, goat anti-mouse Alexa 488 goat anti-rabbit, Alexa 546 at (dilution 1:500, Thermo Fisher Scientific, Waltham, MA, USA )) and goat anti-mouse IgM Alexa Fluor 546 at (dilution 1:500, Thermo Fisher Scientific) were used. As isotype control, rabbit (Abcam) at a final concentration of 5 µg/mL or mouse IgG (BD Pharmingen) at a final concentration of 1 µg/mL, as well as rabbit polyclonal IgG (Abcam) at a final concentration of 2 µg/mL were used. Nuclei were stained with TO-PRO-3 (Thermo Fisher Scientific 1:500) or HOECHST (10 ng/mL). After a final washing step, the glass slides were dried and mounted with ProLong^®^ Gold antifade reagent (Invitrogen). Image acquisition was conducted using an LSM 510 Meta attached to an Axioplan 2 imaging MOT using ZEN software 2008 (LSM 710, Zeiss, Oberkochen, Germany) or a NIKON Ti Eclipse confocal microscope (Nikon, Tokyo, Japan).

### 2.6. Immunoblot Assay

Three to 5 × 10^5^ fibroblasts were seeded on gelatin-coated 10cm petri dishes in DMEM (Lonza, Szabo Scandic) and incubated with different concentrations of FCS, BSA or HSA and FCS + AA. Cells were washed with PBS and collected using a cell scraper, centrifuged and re-suspended in TDLP (triple detergent lysis buffer; 0.1% SDS, 1% NP-40; 0.5% sodium deoxycholate (Sigma Aldrich)) containing protease inhibitors. Extracts were treated with ultrasound (Hielscher) and protein content was determined using a BCA kit (Pierce) and calculated using a BSA standard curve (Biorad). 10 to 30 µg of protein extracts were separated on 12% PAA or 8% PAA gels and blotted onto nitrocellulose membranes (G&E Healthcare, Amersham, UK), blocked with 5% non-fat milk powder (Biorad, Hercules, CA, USA) or 5% BSA (Sigma Aldrich) and incubated with the following primary antibodies: Anti-CD90 polyclonal antibody rabbit (Abcam) or Dianova mAb (AS02) or anti-αSMA (Abcam) or DAKO (1A4), anti-fibroblast surface protein (1B10, Sigma Aldrich), polyclonal anti-vimentin (Santa Cruz), Col1A1 (3G3; Santa Cruz):, β-tubulin rabbit mAb (3F3 Cell Signaling), GAPDH rabbit mAb (14C10; Cell Signaling). Incubation of first antibodies was carried out ON. Secondary antibodies coupled with HRP (Cell Signaling) were incubated for at 1h RT. Blots were incubated with HRP substrate (LiteAblot, Euroclone) and detected by X-ray film (Thermo Fisher Scientific, Waltham, MA, USA). Specific signals were normalized to GAPDH or tubulin using the Image Quant software TL 8.1(GE Healthcare Life Sciences, Marlborough, MA, USA).

### 2.7. XTT Assay for Determination of Cell Viability

Cells were grown for 24 h on gelatin-coated 96 well plates. XTT sodium salt powder (Biomol, Hamburg, Germany) was dissolved in medium according to the manufacturer’s instructions and phenazine methosulfate (PMS) (at 5 µM) was added. The plates were incubated for an additional 4 h and the optical density was measured at 450 nm (reference wavelength –595 nm) using a Victor^3^ ELISA reader (Perkin Elmer, Waltham, MA, USA). For quantifications, the background levels of media without cultured cells were subtracted.

### 2.8. Sirius Red staining of Fibroblasts

Fibroblasts were grown on glass chamber slides (Corning) for 24 h. Afterwards cells were FCS starved. Cells were incubated with different amounts or no FCS (0, 0.5, 1, 2, 4, and 10%), with or without 100 µM AA. Supernatants were frozen at −80 °C until further use. Cells were stained with Sirius Red (SR) according to [33]. Briefly, cells were fixed with methanol ON at −20 °C and carefully washed with PBS and stained with 1% picro-Sirius red solution (Sigma) for 1h at RT. Cells were washed with 0.1% acidic acid, treated with ethanol followed by xylene and mounted with Entellan (Merck, Darmstadt, Germany). Image acquisition was conducted using a Nikon C2 Eclipse microscope.

### 2.9. Collagen 1 ELISA

Supernatants of cell culture (48 h) after incubation with different concentrations of FCS as described above and with AA (100 µM), were frozen at −80° prior to further analysis. Supernatants were diluted 1:20 and collagen 1 was quantified with human procollagen I alpha 1 (COLIA1) ELISA according to the manufacturer’s instructions (R&D Systems). The optical density was measured using a TECAN Spark reader and results were calculated by subtraction of 450–540nm values.

### 2.10. Statistical Analysis

Statistical analyses were performed using GraphPad Prism 6-software (GraphPad, San DiegoLa Jolla, CA, USA). Groups were compared using one-way ANOVA and Tukey’s multiple comparison test. *p* values < 0.05 were considered as statistically significant.

## 3. Results

### 3.1. CD90 + Cultured Primary Human Fibroblasts Express αSMA

**Figure 1:**
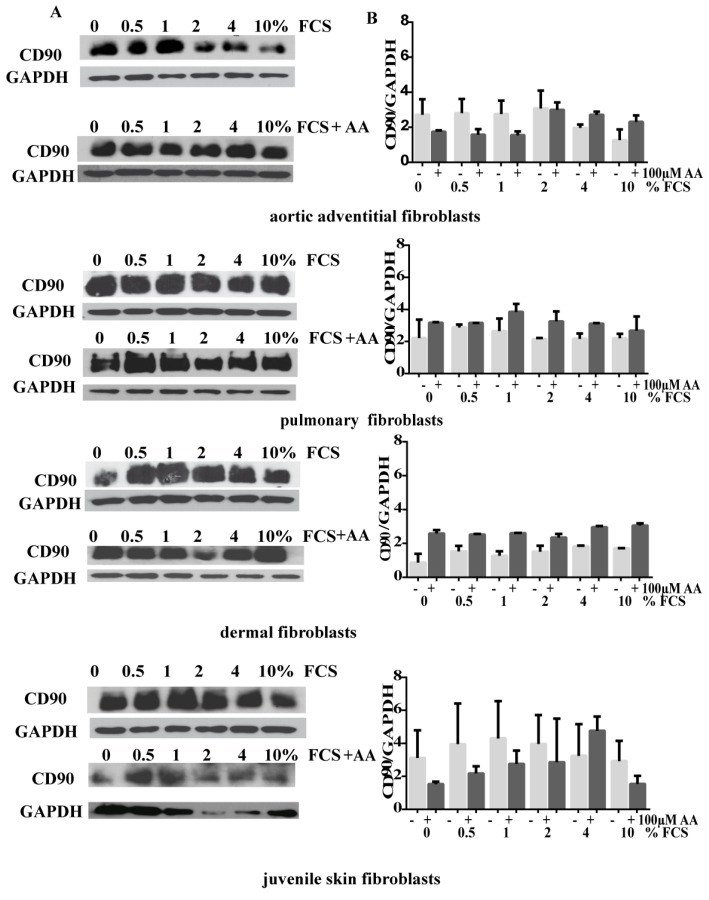
Expression levels of CD90 with different concentrations of fetal calf syndrome (FCS) and FCS plus 100 µM ascorbic acid (AA) within the culture medium of different fibroblast types. (**A**) Expression levels of CD90 are illustrated by immunoblots as indicated. For normalization, protein levels of GAPDH are demonstrated. (**B**) Quantification of CD90 is present as ratio of CD90/GAPDH signal. Data are the mean of at least 2–4 experiments with polyclonal and monoclonal Abs for CD90.

To assess if different concentrations of heat inactivated FCS influence primary human fibroblasts in vitro, CD90+ cells were isolated from the adventitial layer of ascending aortic tissue and then cultured in medium with increasing FCS concentrations for 48 h. For comparison, fibroblasts from dermal fat tissue, from juvenile fore skin and from adult lung were isolated. Cells were cultured at early passages with different amounts of FCS supplemented to media (without FCS up to 10% as indicated). Additionally, the effect of AA on fibroblasts of different origin in combination with increasing amounts of FCS was studied. We observed a decrease in the CD90 expression only in human adventitial aortic fibroblasts (AoAF) with increasing amounts of FCS (Figure 1). CD90 expression levels seems to be lower with none or low amounts of FCS and AA, but increases with higher concentrations of FCS (Figure 1) in human adventitial fibroblasts.

Pulmonary CD90+ fibroblasts did only slightly change their CD90 expression, while adult dermal fibroblasts showed increased CD90 expression levels in response to higher amounts of FCS and AA (Figure 1B).

**Figure 2:**
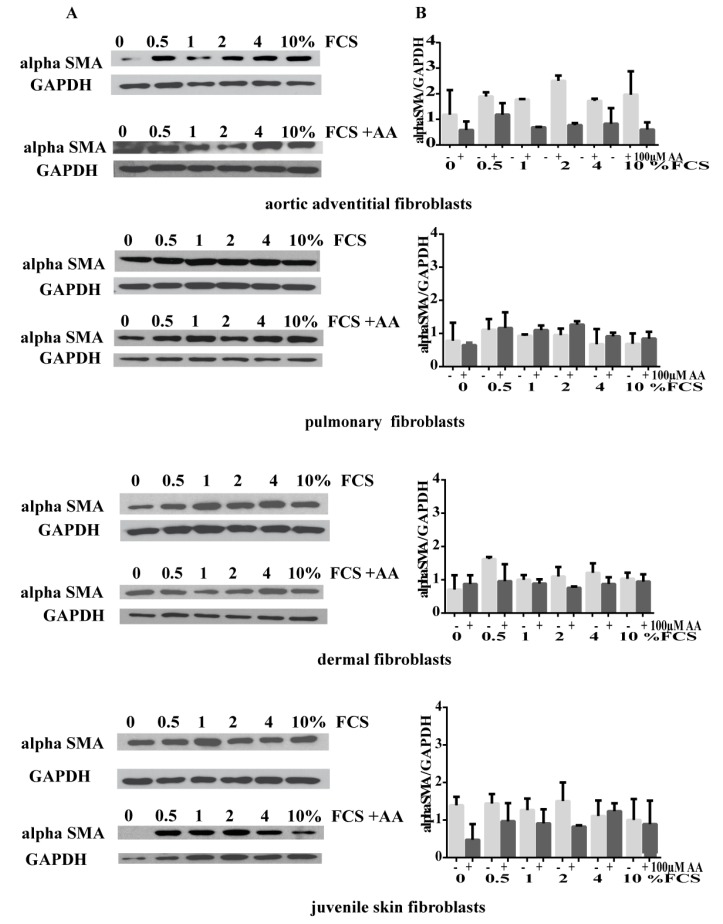
Expression levels of alpha SMA with different concentrations of FCS and FCS plus 100 µM Ascorbic acid in culture medium in fibroblasts. (**A**) Expression levels of αSMA are illustrated by immunoblots as indicated. For normalization, protein levels of GAPDH are demonstrated. (**B**) Quantification of αSMA is present as ratio of αSMA/GAPDH signal. Data are the mean of at least 2–4 experiments with polyclonal and monoclonal Abs for αSMA.

The expression of αSMA, an early feature for myofibroblast phenotype switching under certain conditions [10,14] was additionally determined in cultured CD90+ fibroblasts. We observed expression of αSMA in all primary cultures of fibroblasts, however the amount of αSMA expression varied with increasing concentrations of FCS (Figure 2). In AoAF, the expression of αSMA was elevated with concentrations of 2% FCS while in pulmonary and skin fibroblasts 0.5% of FCS showed elevated levels of αSMA expression (Figure 2). Interestingly, all fibroblast cultures expressed αSMA without FCS as a supplement. Addition of AA did not reverse the effect of myofibroblast differentiation in primary fibroblasts, however in AoAF and juvenile skin fibroblasts without FCS protein levels of αSMA were reduced and did not change with different amounts of FCS in combination with AA.

**Figure 3:**
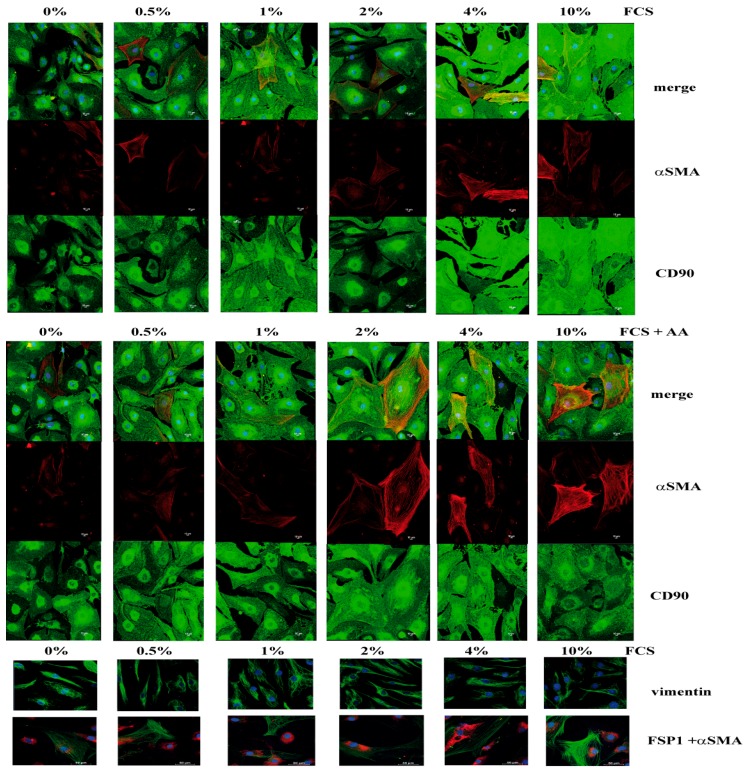
Expression levels of CD90 (green), FSP1 (red), vimentin (green) and αSMA (green) in AoAFs shown by immunofluorescence staining and confocal microscopy pictures. CD90 (Thy-1), FSP1, and vimentin are expressed in all fibroblasts, while αSMA is expressed only in individual cells. Nuclei were stained with TOPRO-3 or Hoechst-stain and are shown in blue.

To further investigate the frequency of αSMA-expressing fibroblasts, we performed immunofluorescence staining and confocal microscopy for the different types of fibroblasts. While all cells expressed CD90, only some cells within the same culture and mainly AoAF co-expressed αSMA. Furthermore, the αSMA expression was increased at higher FCS levels ± AA as illustrated for AoAF (Figure 3).

Vimentin expression was demonstrated in all cells and expression levels were not changed under different FCS concentrations. Additionally, fibroblast surface protein (FSP1) did not show any differential expression patterns in immunofluorescence-based analysis (Figure 3). In contrast, in confocal microscopy pulmonary fibroblasts showed only marginal expression of αSMA at low levels of FCS which was not further induced by high dose FCS (data not shown). Fibroblasts of adult and juvenile skin showed an FCS independent αSMA expression pattern of only few cells and a constant vimentin and FSP1 expression pattern (data not shown).

### 3.2. Increasing FCS Concentration in addition with AA Changes the Viability and Reduces the Migration Capability of AoAF and Other Fibroblast Types

**Figure 4:**
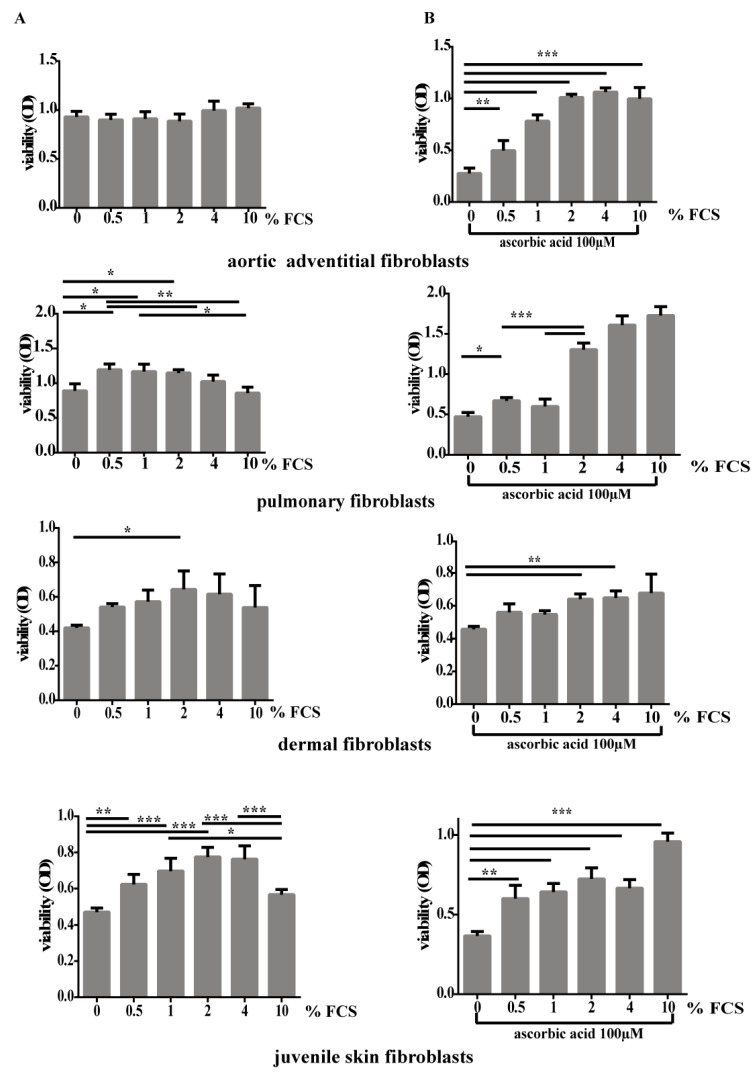
Determination of viability in different fibroblast types with increasing FCS concentrations with or without AA. (**A**) Incubation of AoAF, lung, adult dermal and juvenile skin fibroblasts with different concentrations of FCS increases viability, with the exception of AoAFs. (**B**) in contrast incubation of cells with AA without FCS reduces the cell viability, but addition of FCS increases viability to normal levels. Columns and error bars represent the mean and SD of optical density. * *p* < 0.05, ** *p* < 0.01, *** *p* < 0.001.

To determine the viability of fibroblasts we performed a metabolic activity assay (XTT assay) (Figure 4A).

AoAF showed no change in viability with increasing concentrations of FCS (Figure 4A). In contrast, dermal fibroblasts (both juvenile and adult skin fibroblasts) showed an optimal metabolic activity at 2–4% FCS while pulmonary fibroblasts exhibited increased viability at 0.5% FCS (Figure 4A). Addition of AA to low amounts of FCS or no FCS, reduced the viability especially in AoAF, however higher doses of FCS resulted in similar viability as with FCS alone (Figure 4B).

**Figure 5:**
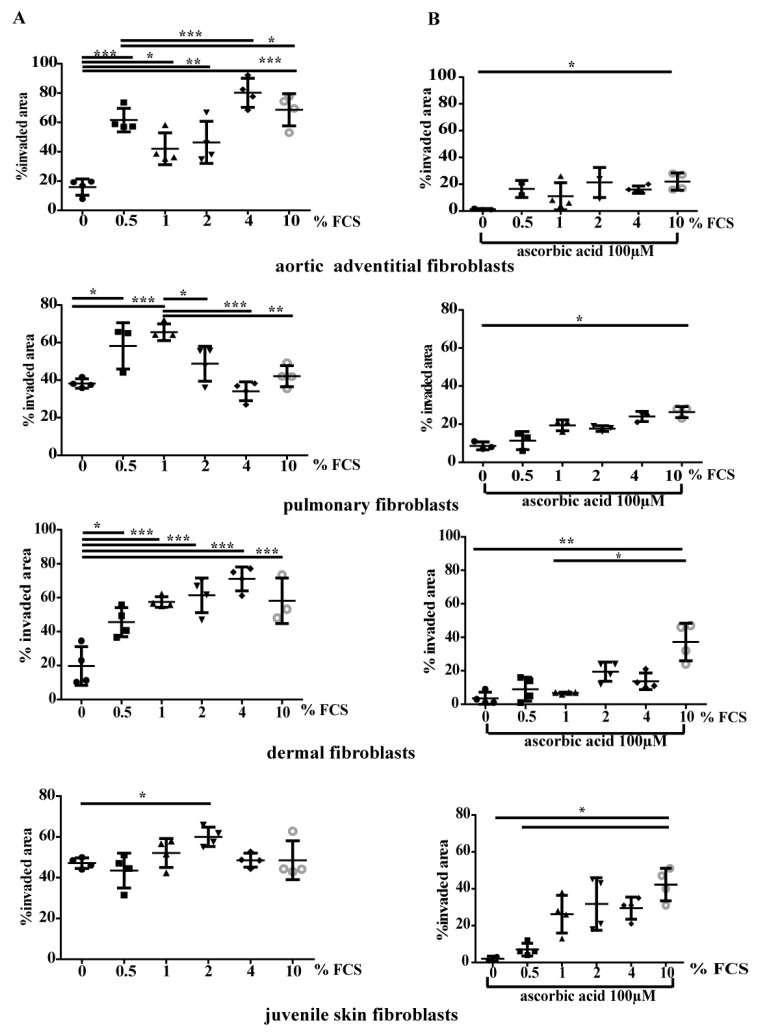
Migration of fibroblasts is reduced by addition of AA to FCS. (**A**) Migration of different fibroblast types incubated with different concentrations of FCS is demonstrated as % invaded area in scatter blots. In (**B**) migration with additional AA to different FCS concentrations is shown. * *p* < 0.05, ** *p* < 0.01, *** *p* < 0.001.

Migration was determined in all fibroblast types using a wound healing assay (in vitro scratch assay) (Figure 5). Pulmonary and juvenile skin fibroblasts had improved migration properties than AoAF and adult dermal fibroblasts without addition of FCS to culture media. Interestingly, juvenile skin fibroblast showed a significant increase in migration only with an FCS concentration of 2%. AoAF showed improved migration at higher concentrations of FCS (4 and 10% of FCS). Pulmonary fibroblasts, however, migrated best at 1% and 0.5% FCS concentration in cell culture media. Furthermore, adult dermal fibroblasts had the best migratory properties with 4% FCS added to cell culture media (Figure 5A). Addition of AA to different concentrations of FCS resulted in a reduced migration capability (Figure 5B).

### 3.3. Minor Effects of Bovine or Human Serum Albumin on the Myofibroblast Phenotype Switch in Primary Human Fibroblasts

TGF-β1 was described to be detectable in an inactive form in FCS [34]. Therefore, we hypothesized that TGF-β1 is not responsible for myofibroblast transition in cell culture in this setting. Since bovine albumin (BSA) is the most abundant protein in FCS [31] and human albumin (HSA) in human serum, we investigated if different amounts of albumin could influence the myofibroblast phenotype of cultured fibroblasts in vitro. Albumin levels were adapted to the different amounts of FCS and added to media at increasing concentrations. Expression levels of αSMA did not differ when BSA or HSA were added to cell culture media (Figure S1). In XTT assays, the cell viability did not change with HSA and BSA as a supplement (Figure S2). Additionally, in migration assays only AoAF improved their migration ability in response to higher albumin concentrations whereas other fibroblast types showed non-significant fluctuations in migration with increasing amounts of albumin (Figure S3). Therefore, albumin does not seem to promote the myofibroblast-like phenotype in primary fibroblast cultures in vitro.

### 3.4. Intracellular Procollagen I Accumulation Differs with AA and Varying FCS Concentrations

Additionally to αSMA, type I and type 3 collagens were described to be expressed in cells of myofibroblast phenotype after TGF-β1 stimulation of rodent kidney fibroblasts [17,35]. To estimate the collagen content within the different primary fibroblasts from different organs, we performed Sirius red staining (Figure S4). Intracellular collagen seemed to be accumulated in AoAF with increasing amounts of FCS and AA. This was also visible in the pulmonary and skin fibroblasts. In fibroblasts growing in media with higher amounts of FCS however, characteristic nodules were visible indicating secretion of collagen (Figure S4 black arrows).

**Figure 6:**
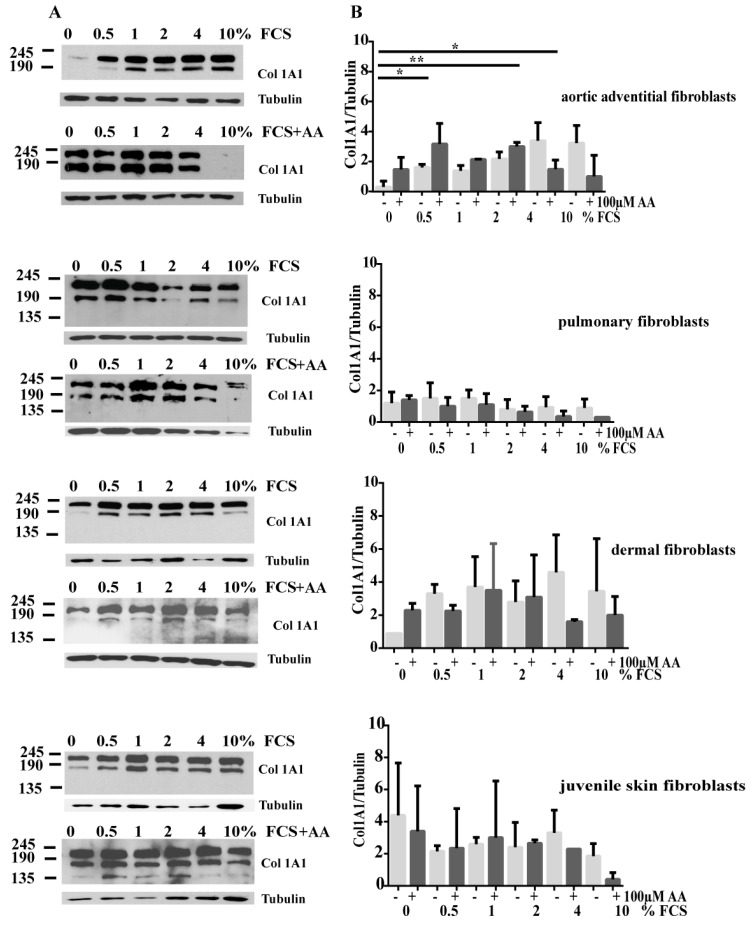
Procollagen I accumulates with increasing amounts of FCS in AoAF and skin fibroblasts, but is secreted after addition of AA. (**A**) Procollagen I (Col 1A1) expression after incubation with different amounts of FCS or without FCS is demonstrated in immunoblots. Tubulin is demonstrated as loading control. (**B**) Procollagen I expression in the presence of AA and with different amounts of FCS is demonstrated in immunoblots.

To elucidate differences in procollagen I expression, indicating a myofibroblast-phenotype, we performed immunoblots with procollagen I-specific antibody. In whole cell extracts of AoAFs high amounts of procollagen I were detectable with increasing doses of FCS (Figure 6). Additionally procollagen I expression was decreased in AoAF with increasing amounts of FCS and AA, suggesting that higher amounts of collagen might be secreted (Figure 6).

**Figure 7:**
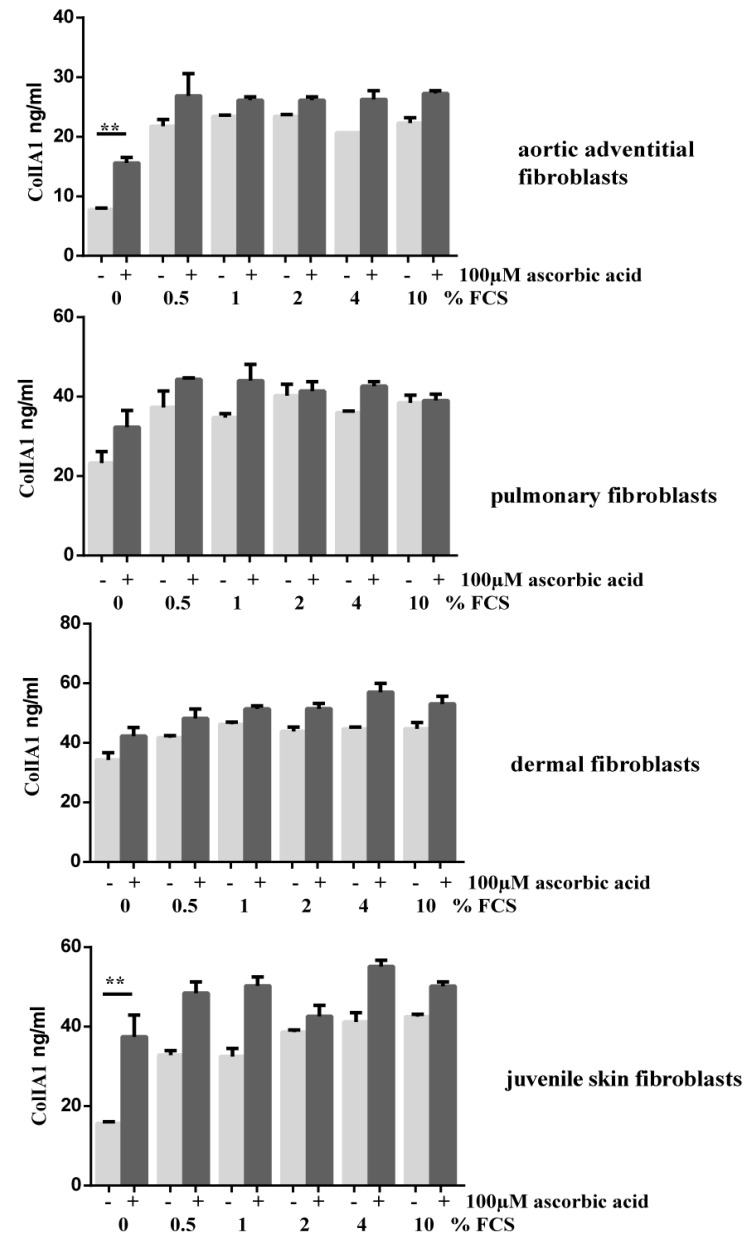
Ascorbic Acid enhances secretion of collagen IA1 in primary fibroblasts with different concentrations of FCS or without FCS. Secretion of collagen IA1 was quantified by ColIA1 specific ELISA: Amounts are demonstrated in ng/mL as columns with standard deviation. ** *p* < 0.01.

The same effect was visible in dermal fibroblasts (Figure 6). Pulmonary fibroblasts, however reacted differently and showed less intracellular procollagen I expression at higher serum levels (Figure 6). In cell culture conditions with FCS and AA, intracellular procollagen I accumulation also decreased with increasing doses of FCS (Figure 6). In adult and juvenile skin fibroblasts, an additional band of about 130kDa was detectable in lysates of cells incubated with higher amounts of FCS and AA. This indicates that all three forms of procollagen necessary for conversion of procollagen into collagen are detectable [19]. Since we found accumulation of collagen in Sirius red stained fibroblasts and high expression levels of Col1A1 in cell lysates, we investigated secretion of procollagen 1 of different fibroblasts under different conditions using a procollagen I ELISA. All cell lines released substantial amounts of ColIA1, although the collagen levels secreted were lower in cell cultures without serum. Significantly higher amounts of soluble procollagen 1 were detectable in AoAF and juvenile skin fibroblasts without serum and AA compared to cultures without FCS (Figure 7).

Additionally, cells incubated with FCS and AA mostly showed higher amounts of secreted Col 1A1 in contrast to cells supplemented with FCS alone.

## 4. Discussion

Culture conditions, especially the presence of TGF-β, were demonstrated in several studies to promote transition of fibroblasts to a myofibroblast phenotype. As an additional supplement AA seems to enhance the effect and lead to an increase of collagen type 1 production. Indeed, in cell culture media commercially available and in usual media, FCS as well as other supplements are added in different concentrations, therefore transition of primary fibroblasts to a myofibroblast-like phenotype may vary.

We found that CD90 (Thy 1) expression is differentially regulated in primary fibroblasts with different concentrations of FCS and AA, while addition of TGF-β1 to cardiac fibroblasts was described not to change CD90 expression but myofibroblast contractile markers [36]. Nevertheless, variations in expression levels of CD90 seems not to correlate with the myofibroblast marker αSMA.

The vascular adventitia is a critical mediator in vessel remodeling and arterial disease progression as well as after vascular intervention and in vascular inflammation initiation. Consequently, adventitial fibroblasts (AF) become activated. Excessive collagen deposition leads to thickening and stiffening of vessel wall and supports vascular fibrosis, which influences the AF phenotype [37,38]. Additionally, AF play an important role in vessel remodeling through their increased migration ability [39,40]. Therefore, to obtain optimal secretion of collagen 1 the addition of FCS and AA seems to be more efficient than with FCS alone. Secretion of procollagen I especially with moderate or higher amounts of FCS seems to be more efficient in all primary fibroblasts tested.

Albumin is often used as a serum replacement for different cell culture conditions. Since the most abundant protein in serum is albumin, we challenged cultured fibroblasts of different origin with increasing amounts of BSA, as a xeno-protein, and HSA, the human albumin. Purified albumin, independently if bovine or human albumin was used, however had only minor effects on myofibroblast differentiation. In our study, higher amounts of BSA even showed reduction of viability in some primary fibroblast lines. Notably, purified albumin is a supplement often used in serum-free media commercially available [41].

Medium supplements have an important influence on growth performance as exemplified for the proliferation and differentiation of mesenchymal stem cells [42]. Cultured primary fibroblasts behave differently depending on their origin. Interestingly, only a part of the cells differentiates to a myofibroblast-like phenotype depending on the culture conditions. This effect can partially be suppressed with addition of AA. Additionally, AA supplemented to primary fibroblasts in culture leads to secretion of procollagen I rather than intracellular accumulation with higher amounts of FCS Furthermore, even though not addressed in this study, it is highly feasible that fibroblasts isolated from diseased tissue may also have disorder-associated features in culture compared to primary cells from healthy donors. Differences in age of the original tissue should also be considered in cultured primary cells. All these parameters have to be evaluated regarding in vitro model systems based on cell culture. Most striking, the mere in vitro culture of fibroblasts (irrespective of media composition, serum inclusion) seems to trigger the differentiation to the myofibroblast phenotype.

## 5. Conclusions

Primary cell types are rare and important for disease-related in vitro studies as opposed to immortalized cell lines. However, cell culture conditions, in particular the addition of supplements to media can change the phenotype of primary cells in vitro.

## Supplementary Materials

The following are available online at https://www.mdpi.com/2073-4409/8/7/721/s1, Figure S1 to Figure S3: Additional data with different amounts of BSA and HSA respectively. Figure S4: Sirius red staining for estimation of collagen with different culture conditions in primary human fibroblasts.
