# Supplementary material for: Primary Human Fibroblasts in Culture Switch to a Myofibroblast-Like Phenotype Independently of TGF Beta
Source: Cells. 2019 Jul 13;8(7):721. doi: 10.3390/cells8070721 (PMC6678602; doi:10.3390/cells8070721)
Supplement: Supplementary file 1 [file cells-08-00721-s001.pdf]

Supplementary figures

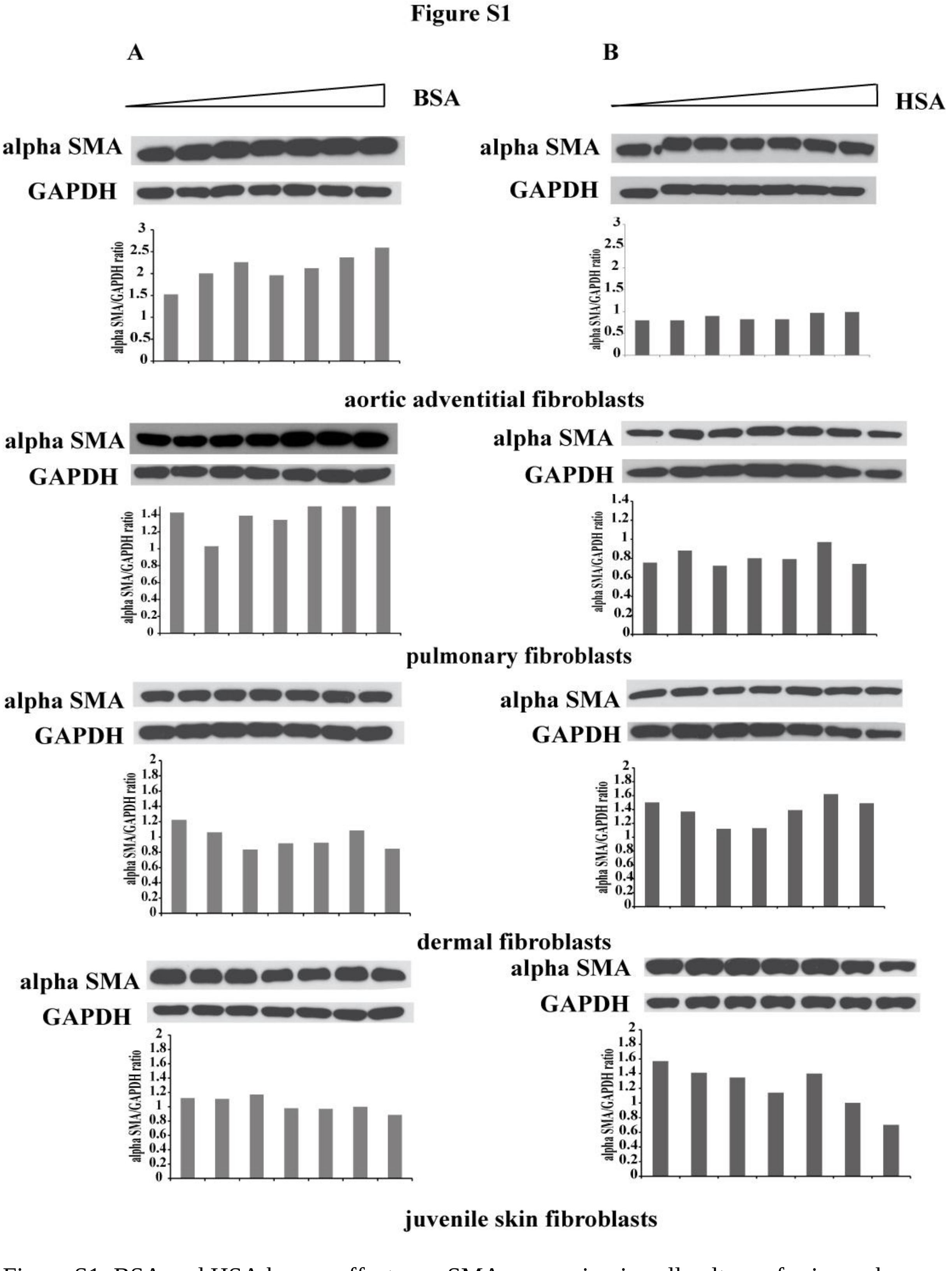


Figure S1: BSA and HSA has no effect on αSMA expression in cell culture of primary human fibroblasts. Expression levels of αSMA are illustrated by immunoblots and normalized to

GAPDH expression.

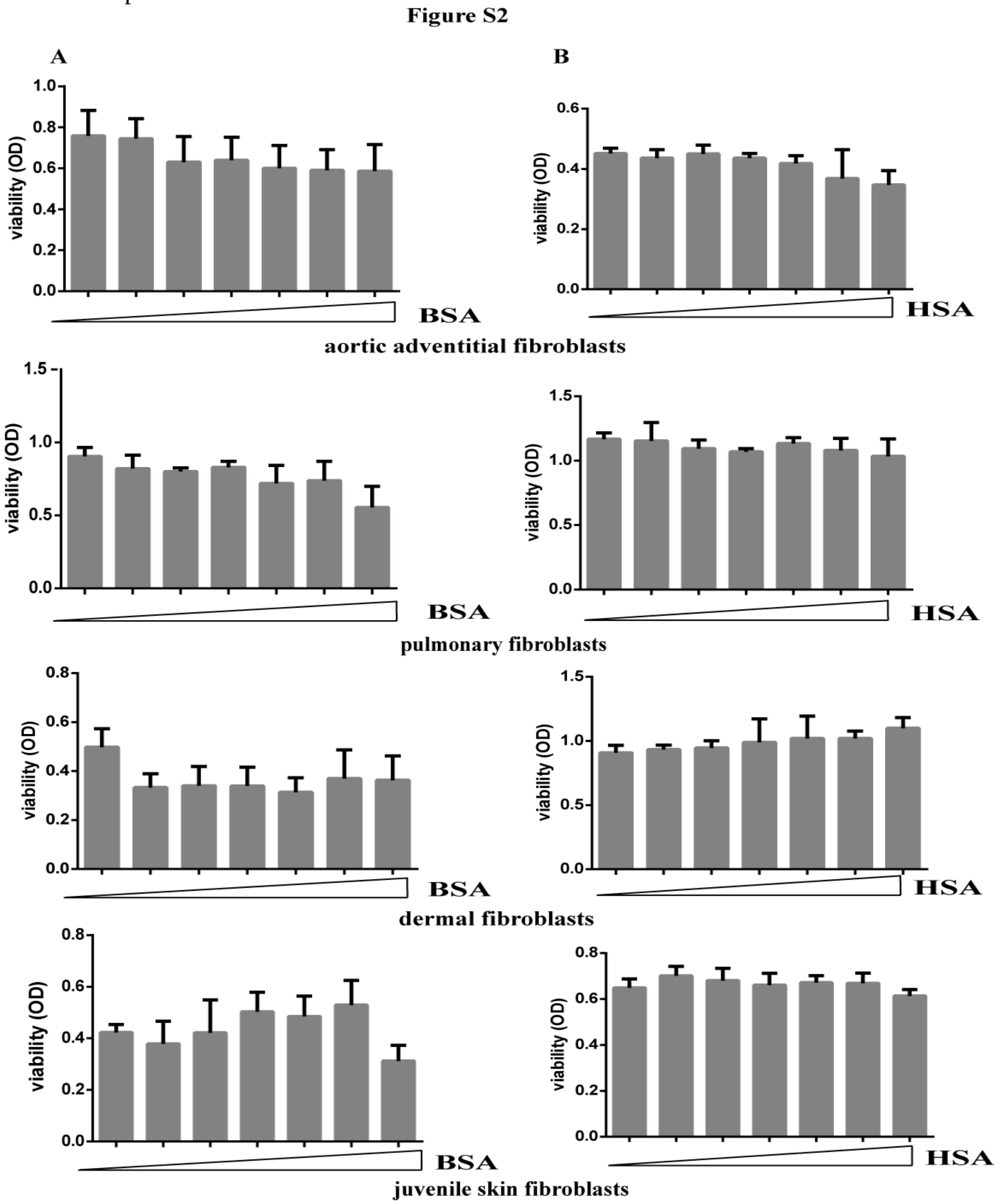


Figure S2: Viability of human fibroblasts is not influenced by serum albumin. Viability of primary fibroblasts is demonstrated with increasing levels of BSA (left panel) or with increasing levels of HSA respectively (right panel). Columns and error bars represent the mean and SD of optical density.

**Figure S3**

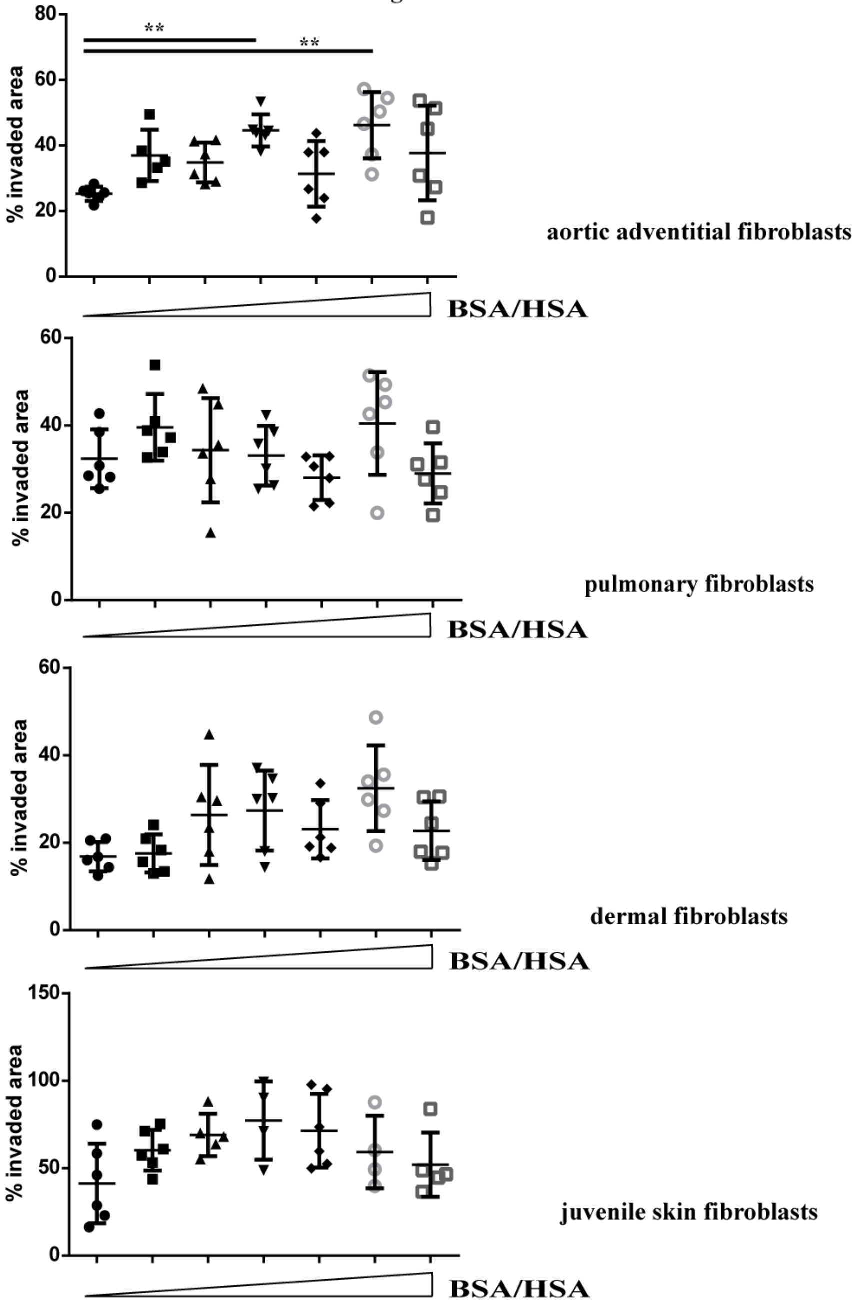


Figure S3: Migration changes marginally for fibroblasts cultured with different concentrations of albumin. Percent of invaded area in scratch assays of different primary fibroblast types is demonstrated in scatter plots for increasing concentrations of BSA and HSA (0.0115%, 0.023%, 0.046%, 0.092%, 0.23% and 0.46%) in culture medium. ** $p<0.01$

## Figure S4

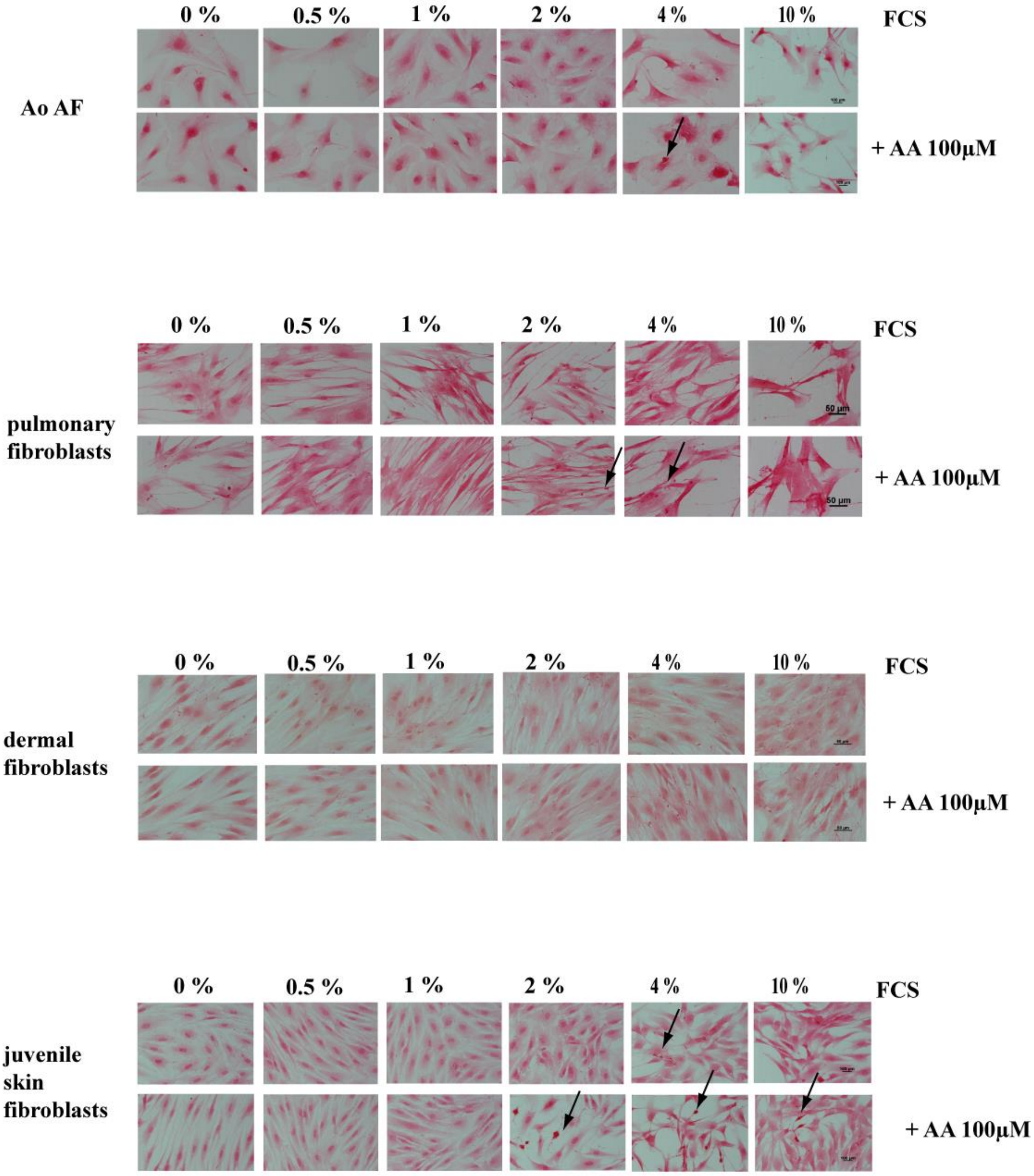


Figure S4: Fibroblasts are accumulating collagen with increasing amounts of FCS. Sirius red stained cells show intracellular collagen and signs for secretion indicated by arrows. Light microscopic pictures are demonstrated.
